# The neural stem cell gene *PAFAH1B1* controls cell cycle progression, DNA integrity, and paclitaxel sensitivity of triple-negative breast cancer cells

**DOI:** 10.1016/j.jbc.2025.110235

**Published:** 2025-05-14

**Authors:** Parth R. Majmudar, Ruth A. Keri

**Affiliations:** 1 Department of Pharmacology, Case Western Reserve University School of Medicine, Cleveland, Ohio, United States; 2 Department of Cancer Biology, Lerner Research Institute, Cleveland Clinic, Cleveland, Ohio, United States

**Keywords:** PAFAH1B1, LIS1, breast cancer, triple-negative breast cancer, TNBC, mitosis, DNA damage, paclitaxel, taxane, neural

## Abstract

Triple-negative breast cancer (TNBC) is a highly aggressive disease with limited approved therapeutic options. The rapid growth and genomic instability of TNBC cells make mitosis a compelling target, and a current mainstay of treatment is paclitaxel (Ptx), a taxane that stabilizes microtubules during mitosis. While initially effective, acquired resistance to Ptx is common, and other antimitotic therapies can be similarly rendered ineffective due to the development of resistance or systemic toxicity, underscoring the need for new therapeutic approaches. Interrogating CRISPR essentiality screens in TNBC cell lines, we identified *PAFAH1B1* (LIS1) as a potential vulnerability in this disease. *PAFAH1B1* regulates mitotic spindle orientation, proliferation, and cell migration during neurodevelopment, yet little is known regarding its function in breast cancer. We found that suppressing *PAFAH1B1* expression in TNBC cells reduces cell number, while non-malignant cells remain unaffected. *PAFAH1B1* suppression alters cell cycle dynamics, increasing mitotic duration and accumulation of cells in the G2/M phase. The suppression of *PAFAH1B1* expression also increases DNA double-strand breaks, indicating a requirement for sustained *PAFAH1B1* expression to maintain the genomic integrity of TNBC cells. Finally, *PAFAH1B1* silencing substantially enhances these defects in cells that are taxane-resistant and sensitizes both parental and Ptx-resistant TNBC cells to Ptx. These results indicate that LIS1/*PAFAH1B1* may be a novel target for the development of new anti-mitotic agents for treating TNBC, particularly in the context of paclitaxel resistance.

Triple-negative breast cancer (TNBC) is the most aggressive subtype of breast cancer. While it accounts for only 10 to 15% of cases, it has the lowest 5-year survival rate, especially for regional or distant disease (1). This is due, in part, to higher rates of proliferation and metastasis (2). Given its high degree of heterogeneity, targeted therapies that are universally effective for all patients with TNBC have remained elusive. Rather, small subsets of patients whose tumors meet specific criteria are treated with targeted agents (3). Because of the paucity of targeted therapies, most patients with TNBC are treated with cytotoxic chemotherapies such as taxanes, which leverage the intrinsic chromosome instability of this disease to cause cell death (4). Despite being initially effective and inducing pathologic complete responses in a subset of patients, many tumors develop resistance, likely reflecting the presence and subsequent expansion of small populations of non-responder or persister cells (5). As a result, patients with TNBC experience higher relapse rates and worse survival outcomes, emphasizing the need for new drug targets that are broadly necessary for the growth and maintenance of tumor cells.

Breast cancer cells often misappropriate pathways and processes involved in normal mammary gland morphogenesis (6). Several groups have worked to identify the cell-of-origin of different subtypes of breast cancer, linking tumor expression patterns to cell types found in the developing gland. Subsets of TNBC often exhibit transcriptomic profiles similar to mammary stem and progenitor cells, while Luminal A and B cancers align more closely to mature luminal cells (7, 8). The earliest stages of mammary epithelial development involve the formation of the mammary line from the ectoderm, the outermost layer of germ cells that also gives rise to the neuroectoderm, the precursor of the central and peripheral nervous systems (9). Notably, TNBC tumors and cell line models express many neural genes at a level that is higher than other subtypes of breast cancer, suggesting that these tumors may activate pathways that sit at the intersection between epidermal and neural cell fate determination (10, 11). TNBCs also exhibit a higher degree of stemness, both functionally and at the transcriptome level, compared to the other subtypes. Taken together, these observations suggest that TNBCs hijack signaling pathways from early developmental states to retain characteristics of stem and progenitor cells and provide a level of adaptability that is less frequently observed in more differentiated breast cancer subtypes.

Considering the ectodermal origins of the mammary epithelium and its close lineal association with neuroectoderm, we sought to identify neural signature genes that may also drive TNBC proliferation. Using a stepwise filtering approach, we interrogated CRISPR essentiality screens in breast cancer cell lines for genes that were classified as a dependency in at least 90% of breast cancer cell lines and were also associated with both neural and mitotic processes. This yielded a final list of three candidate genes: *ANKLE2*, *PAFAH1B1*, and *WEE1*. *ANKLE2* has previously been reported as a promoter of tamoxifen resistance in breast cancer cells, and *WEE1* has been extensively studied as a therapeutic target across multiple cancer types, including breast (12, 13, 14). In contrast, the impact of *PAFAH1B1* on breast cancer cell growth, viability, or therapeutic response has not been explored.

*PAFAH1B1* (Platelet-Activating Factor Acetylhydrolase 1b Regulatory Subunit 1) encodes the protein LIS1, short for Lissencephaly-1, reflecting the phenotype that occurs in humans and mice with its loss of function. Homozygous loss of *Pafah1b1* is embryonically lethal in mice, while heterozygous mutants display disorganized or a complete lack of cortical layers in the brain due to abnormal neuronal migration (15). Neural stem and progenitor cell proliferation is also impaired with *Pafah1b1* loss, implicating LIS1 as a core regulator of multiple processes crucial for neural development (16). In humans, point mutations occurring in *PAFAH1B1* are haploinsufficient, causing lissencephaly, a neurodevelopmental disorder characterized by a smooth brain surface due to the absence of gyri and sulci (17, 18). These mutations often occur in the WD repeat domains and disrupt protein folding, half-life, intracellular localization, and/or interactions with binding partners (19, 20). A key binding partner of LIS1 is dynein and disruption of this interaction leads to the aforementioned phenotypes (21). Dynein plays an essential role in many cellular processes, including the intracellular transport of vesicles and organelles as well as microtubule spindle organization and chromosome movement during mitosis. Much of the function of dynein is regulated by a host of binding partners, including LIS1, NDE1, NDEL1, BICD2, ZW10, Spindly, and the protein complex dynactin (22). Genetic knockout or mutation of one or more of these partners can disrupt dynein functionality by impeding its recruitment, processivity, attachment, or movement (22, 23, 24). Given its importance in mitosis, dynein suppression is a therapeutic strategy for combating highly proliferative cancers such as TNBC. However, given its participation in numerous intracellular processes in most cells, targeting one of its regulatory binding partners may offer potent antitumor benefits while minimizing potential off-target effects. In this regard, LIS1 is the only known regulator that binds directly to the dynein motor domain to relieve dynein auto-inhibition (25).

*PAFAH1B1/*LIS1 has been reported to be both pro- and anti-oncogenic. In liver cancer, *PAFAH1B1* mRNA is often downregulated and acts as a tumor suppressor, with its overexpression leading to reduced proliferation (26). However, increased *PAFAH1B1* expression is also associated with malignant phenotypes in most cancer models studied, including glioblastoma, non-small cell lung cancer, leukemia, cholangiocarcinoma, and salivary gland carcinoma (27, 28, 29, 30, 31). These apparently contrasting phenotypes are compatible with the role of LIS1 in controlling chromosome segregation where excessive or reduced expression would be expected to produce similar phenotypes, given the goldilocks nature of many proteins involved in mitosis and development (32). The only study examining LIS1 function in breast cancer reported that it is essential for the transport of MT1-MMP secretion and cellular invasion (33). Thus, our understanding of the function and importance of LIS1/*PAFAH1B1* in breast cancer, particularly during mitosis, is limited.

Herein, we report that LIS1/*PAFAH1B1* is a core driver of proliferation and viability in TNBC. Non-transformed breast epithelial cells are unaffected by *PAFAH1B1* silencing, whereas TNBC cells exhibit reduced cell growth. This is primarily due to abnormal cell cycle progression, with an increased proportion of cells stalled in the G2/M phase that is associated with an increase in DNA double-strand breaks and apoptosis, indicating that LIS1 is a novel regulator of DNA integrity. *PAFAH1B1* silencing causes even greater growth suppression, DNA damage, and apoptosis in cells that have acquired resistance to paclitaxel and improves the potency of Ptx in both parental and resistant cells. Together, these data implicate LIS1/*PAFAH1B1* as a novel therapeutic target in TNBC, particularly in paclitaxel-resistant (PtxR) tumors.

## Results

### *PAFAH1B1* constitutes a vulnerability in breast cancer cells that is associated with patient survival

**Figure 1:**
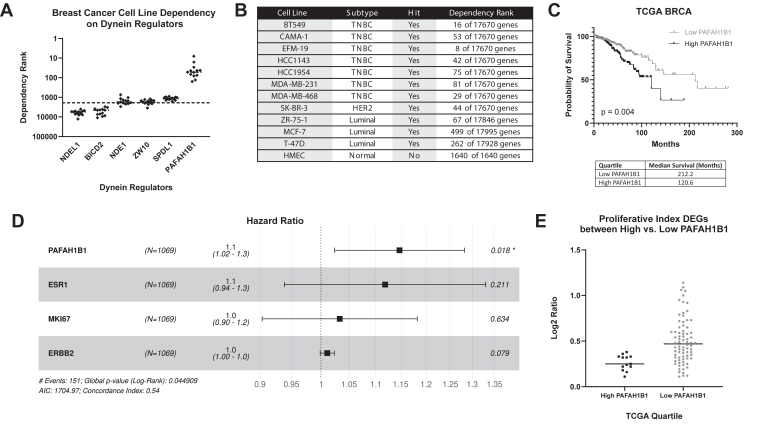
***PAFAH1B1* constitutes a vulnerability in breast cancer cells that is associated with patient survival.***A*, CRISPR essentiality data from Meyers *et al.* shows that breast cancer cell lines were most highly dependent on LIS1 (*PAFAH1B1*) as opposed to other dynein regulators. *Dots* indicate individual cell lines. A lower dependency rank indicates a higher impact of this gene compared to all other genes and is statistically significant if above the dashed line. *B*, ranking of dependencies of TNBC, HER2+, and luminal breast cancer cell lines on *PAFAH1B1*. The normal-like HMEC cell line does not show dependency on LIS1. *C*, Kaplan-Meier analysis of breast cancer patient overall survival when segregated by the upper and lower quartiles of *PAFAH1B1* expression (TCGA, *top* vs. *bottom* quartile, n = 535, *p* < 0.005). *D*, multivariate Cox regression analysis of *PAFAH1B1* association with survival, using *ESR1*, *MKI67*, and *ERBB2* as covariates. *E*, log2FC values of proliferation-associated genes differentially expressed (*p*-adj < 0.05) in the groups of breast cancers constituting the *top* and *bottom* quartiles of *PAFAH1B1* expression. *Dots* indicate individual genes.

Interrogation of publicly available CRISPR essentiality screens for genes that are regulators of neural development and mitosis in breast cancer cell lines revealed *PAFAH1B1* as a candidate driver of breast cancer. *PAFAH1B1*/LIS1 is a well-established modulator of dynein function (34); thus, we determined whether there may be a generalized impact of dynein regulators on breast cancer cell growth. Comparing the dependency rankings for common binding partners of dynein revealed that breast cancer cell lines were consistently far more highly dependent on *PAFAH1B1* than any other dynein regulator (Fig. 1*A*). When considering breast cancer subtype, TNBC cell lines displayed the highest level of dependence on *PAFAH1B1*, with the group of luminal cell lines being modestly less dependent (Fig. 1*B*). Importantly, the single normal human mammary epithelial (HMEC) cell line that was examined did not exhibit dependency. To further explore the association of *PAFAH1B1* with aggressive disease, we interrogated TCGA patient data and found that stratifying breast cancer patients into quartiles by *PAFAH1B1* expression revealed a strong association between higher expression and worse overall survival (Fig. 1*C*). Notably, when accounting for expression of ER (*ESR1*), HER2 (*ERBB2*), and Ki-67 (*MKI67*), *PAFAH1B1* remained associated with worse survival from breast cancer (Fig. 1*D*). More aggressive tumors are highly proliferative; thus, we extended our analysis beyond Ki-67 to assess whether increased proliferation may be driving the worse outcomes of patients whose tumors express elevated levels of *PAFAH1B1*. We used a list of proliferative index genes defined by Venet, *et al.* (35) as a proxy measure of tumor proliferation rate and assessed whether these genes were associated with *PAFAH1B1* expression. This analysis revealed that most differentially expressed proliferation-associated genes were actually more highly expressed in the quartile of tumors with the lowest *PAFAH1B1* expression (Fig. 1*E*). Thus, the association of elevated *PAFAH1B1* in breast cancers with worse patient outcome is not simply dependent on an elevated rate of proliferation within tumors.

### *PAFAH1B1* silencing selectively reduces TNBC cell growth and is associated with enrichment of mitosis-associated pathways

**Figure 2:**
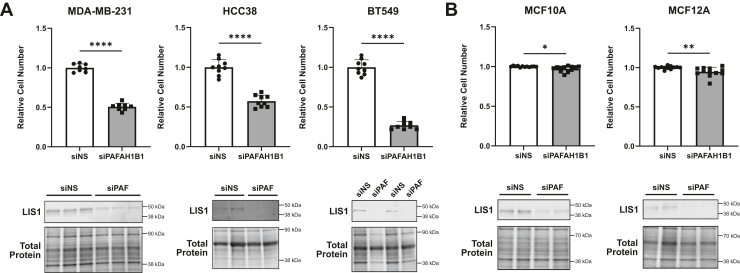
***PAFAH1B1* silencing selectively reduces TNBC cell growth.***A*, TNBC cell lines MDA-MB-231, HCC38, and BT549 were transfected with either non-silencing control siRNA (siNS) or siRNA targeting *PAFAH1B1*, and live cells were counted after 5 days using trypan blue exclusion or quantified using crystal violet staining. *B*, non-malignant breast cancer cell lines, MCF10A and MCF12A, were transfected and growth quantified after 5 days as in panel (*A*). Western blotting was performed at day 5 to confirm *PAFAH1B1* silencing was maintained until the endpoint of the experiment. For all data, n = 3, points are technical replicates for each biological replicate, bars are means ± SD. ∗*p* < 0.05, ∗∗*p* < 0.01, ∗∗∗∗*p* < 0.0001 by unpaired two-tailed *t* test.

To discern the role of *PAFAH1B1*/LIS1 in breast cancer, we focused on TNBC to minimize confounding by different breast cancer subtypes that are driven by distinct signaling pathways (36, 37, 38). According to the Cancer Cell Line Encyclopedia (CCLE), TNBC cell lines (subclassified as Basal A and Basal B) tend to have higher expression of *PAFAH1B1* than luminal breast cancer lines (Fig. S1*A*). Western blotting of a group of breast cancer cell lines confirmed LIS1 protein expression (Fig. S1*B*), and we selected three TNBC lines (MDA-MB-231, BT549, and HCC38) for further analysis. *PAFAH1B1* silencing led to a significant reduction in cell number 5 days after transfection in all three models (Figs. 2*A* and S3*A*). Of note, there was little to no difference in growth 3 days after transfection (data not shown), even though LIS1 protein was effectively suppressed at this time point (Fig. S2). This suggested that TNBC cells are initially able to tolerate defects related to reduced *PAFAH1B1,* but over time, these ultimately become unsustainable. To further explore the selectivity of the effects of *PAFAH1B1* suppression in tumor *versus* non-transformed cells, beyond the single cell line evaluated in the CRISPR screen (Fig. 1*B*), we transiently silenced *PAFAH1B1* in two additional non-malignant cell lines (MCF10A and MCF12A). Neither experienced an appreciable reduction in cell growth in response to *PAFAH1B1* silencing (Fig. 2*B*). These data indicate that *PAFAH1B1* selectively drives breast cancer cell growth without impacting normal mammary epithelial cells.

**Figure 3:**
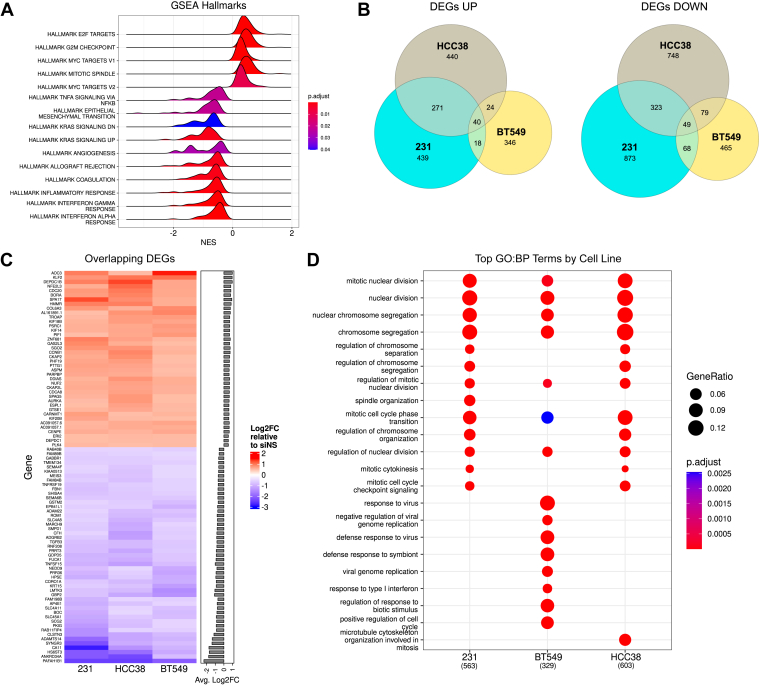
***PAFAH1B1* silencing leads to global changes in the expression of mitosis-associated genes.***A*, MDA-MB-231 cells were transfected with siNS or siPAFAH1B1 in quadruplicate and changes in their transcriptome evaluated by RNA-sequencing. Hallmark gene sets associated with mitosis were enriched in siPAFAH1B1 cells compared to cells transfected with the non-silencing control. *B*, HCC38 and BT549 cells were also transfected with siPAFAH1B1 or siNS in quadruplicate and similarly evaluated by RNA-seq. Venn diagrams are shown for the overlapping DEGs (up and down-regulated) across the three cell lines. *C*, heatmap of the DEGs shared across all three cell lines. *D*, comparison of top GO terms for each cell line individually reveals consistent enrichment of gene sets associated with mitosis and chromosome segregation.

To discover pathways that may mediate the growth suppression of TNBC cells in response to *PAFAH1B1* suppression, we interrogated changes in the transcriptome of MDA-MB-231 cells following transient transfection with siPAFAH1B1 or non-silencing control siRNA (siNS), using RNA-seq. This revealed an enrichment of hallmark gene sets associated with mitosis (Fig. 3*A*). We then validated this finding using RNA-seq analysis of BT549 and HCC38 cells, also with or without *PAFAH1B1* silencing. Overlapping all three data sets identified a consistent set of differentially expressed genes (DEGs) composed of 49 downregulated and 40 upregulated genes (Fig. 3, *B* and *C*). We also assessed the consistency of enriched biological processes from each cell line and found that several of the same pathways were altered with *PAFAH1B1* suppression, albeit with different individual genes being impacted. Gene sets associated with mitosis, specifically nuclear division and chromosome segregation, were consistently overrepresented in all three cell lines (Fig. 3*D*). Thus, *PAFAH1B1* silencing in TNBC cells leads to altered mitotic gene expression that is associated with reduced cell growth.

### *PAFAH1B1* suppression induces G2/M arrest and lengthens mitosis

**Figure 4:**
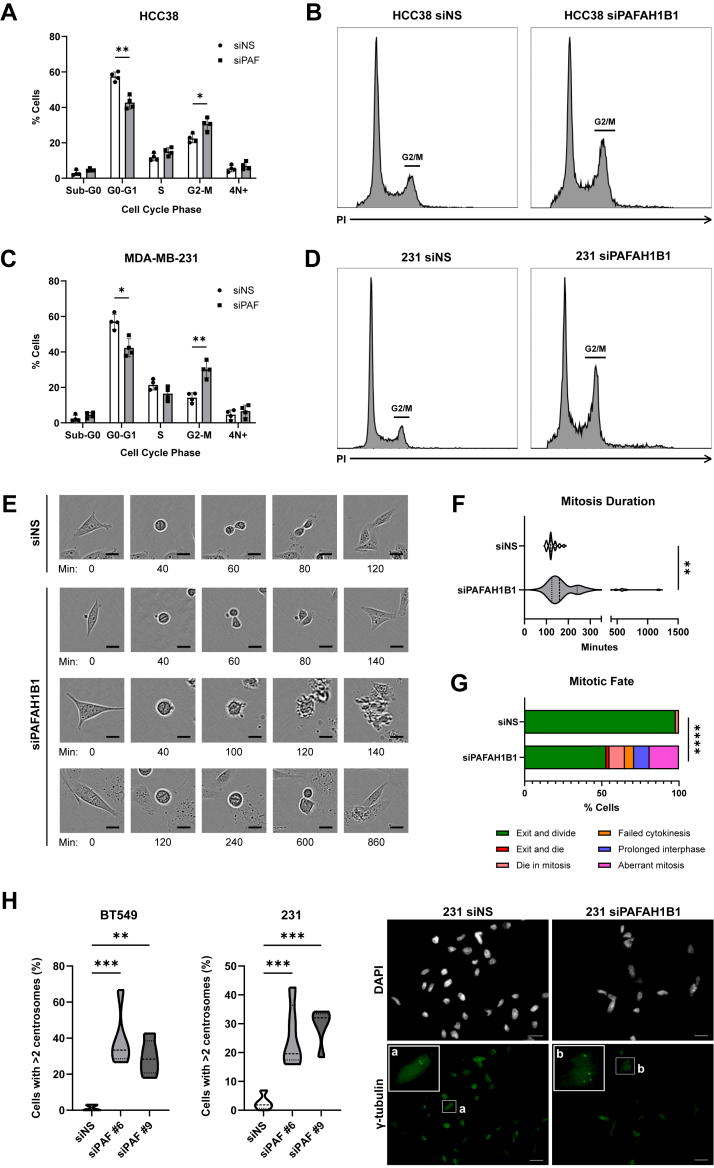
***PAFAH1B1* suppression induces G2/M arrest and lengthens mitosis.***A*, HCC38 cells transfected with siNS or siPAFAH1B1 were fixed and stained with propidium iodide at day three. Flow cytometric analyses revealed a G2/M arrest in *PAFAH1B1*-silenced cells. n = 4, points are biological replicates, bars are means ± SD. *B*, representative PI histograms for HCC38 cells with or without *PAFAH1B1* silencing. *C*, same as panel (*A*), but with MDA-MB-231 cells. n = 4, points are biological replicates, bars are means ± SD. *D*, representative PI histograms for MDA-MB-231 cells with or without *PAFAH1B1* silencing. *E*, live MDA-MB-231 cells transfected with siNS or siPAFAH1B1 were imaged as they traversed mitosis using the Incucyte Live-Cell Analysis System. Images of various mitotic cells over time are shown. Scale bars are 10 μm. *F*, individual cells across multiple fields were manually tracked to assess total time spent in mitosis and values were aggregated. *G*, mitotic fates for all tracked cells were quantified. Expanded definitions for fates are provided in the Methods. Panels (*E–G*) were completed in one technical replicate for which 40 siNS cells and 52 siPAFAH1B1 cells were tracked. *H*, proportion of MDA-MB-231 and BT549 cells with centrosome amplification 4 days after transfection with siNS or siRNAs targeting *PAFAH1B1* (siPAF #6 or #9). n = 3, *dashed lines* indicate quartiles. Representative images of the MDA-MB-231 cells are shown on the right, with labeled insets highlighting cells with normal (*A*) or amplified (*B*) centrosome number. Scale bars are 20 μm. ∗*p* < 0.05, ∗∗*p* < 0.01, ∗∗∗*p* < 0.001 by unpaired two-tailed *t* test for panels (*A*, *C*, *F*, *H*). ∗∗∗∗*p* < 0.0001 by Fisher’s exact test for panel (*G*).

The reduced cell growth and transcriptomic enrichment of mitosis-regulating genes suggested that *PAFAH1B1* suppression may induce defects in cell cycle progression. To test this possibility, propidium iodide (PI) staining coupled with flow cytometry was used to quantify changes in the percentage of cells in each phase of the cell cycle using two different cell lines. Silencing of *PAFAH1B1* resulted in a G2/M arrest in both HCC38 and MDA-MB-231 cells (Fig. 4, *A*–*D*). While not statistically significant, we also noted a slight increase in the sub-G0 and 4N+ populations, representing dead cells and cells with increased DNA content, respectively. The gating restrictions used in these experiments may preclude observing an increase in the sub-G0 population. In contrast, the clear increase in the number of cells in G2/M, and the enrichment of mitotic expression signatures described above, suggested that *PAFAH1B1* may control mitotic progression. To directly assess the impact of *PAFAH1B1* suppression on mitosis, live MDA-MB-231 cells that had been transfected with siPAFAH1B1 or siNS were evaluated as they progressed through mitosis (Fig. 4*E*). As a group, cells with *PAFAH1B1* silencing experienced mitotic delays and exhibited a variety of mitotic fates, with only some successfully exiting mitosis (Fig. 4, *F* and *G*). Comparing the fates of these cells against siNS-transfected cells revealed a 5-fold increase in the proportion of cells that died during or immediately after mitosis (Fig. 4*G*). In addition, an expanded subset of cells exhibited aberrant mitoses, such as disorganized metaphase plates and chromatin bridges, suggestive of multipolar spindles. These defects are often the result of centrosome amplification that can lead to improper chromosome segregation and DNA damage. Indeed, suppressing *PAFAH1B1* resulted in centrosome amplification in both MDA-MB-231 and BT549 cells (Fig. 4*H*). These data indicate that sustained expression of *PAFAH1B1* in TNBC cells is essential for normal mitotic progression.

### *PAFAH1B1* suppression induces DNA double-strand breaks

**Figure 5:**
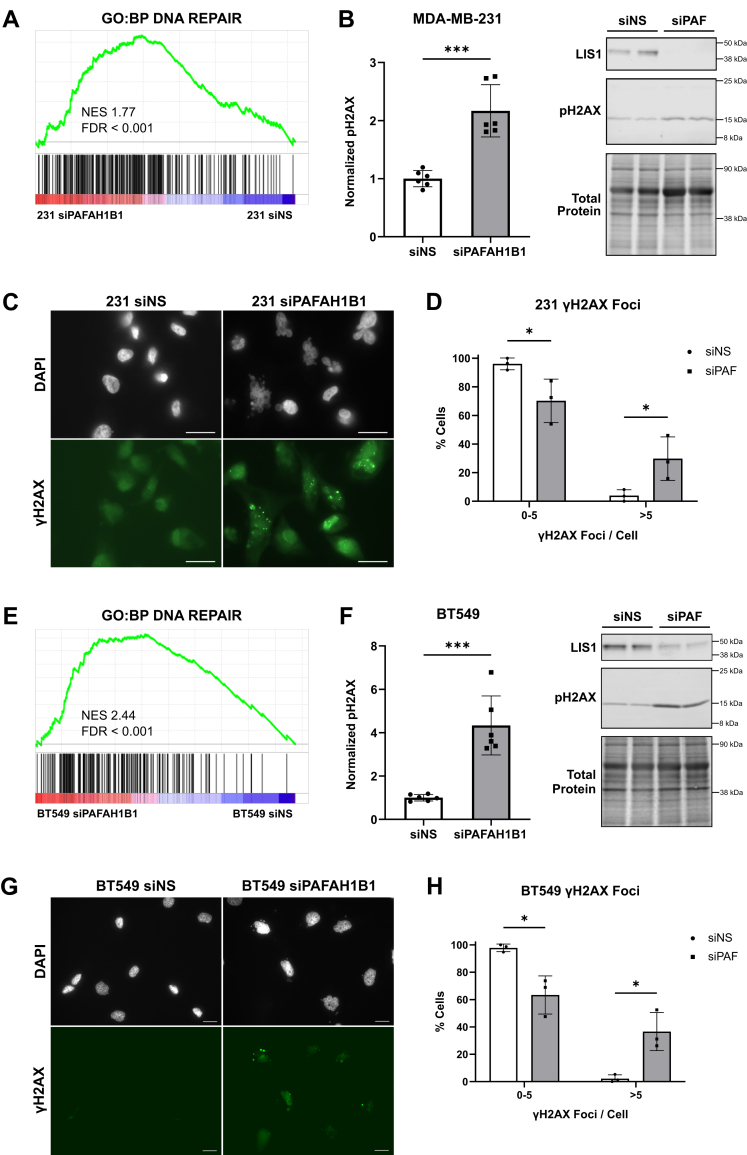
***PAFAH1B1* suppression induces DNA double-strand breaks.***A*, GSEA of RNA-seq data from MDA-MB-231 cells 3 days after transfection with siPAFAH1B1 *versus* siNS reveal enrichment of the DNA repair gene expression signature. *B*, Western blotting for pH2AX expression in MDA-MB-231 cells 5 days after transfection with siPAFAH1B1 or siNS. *C*, MDA-MB-231 cells transfected with siNS or siPAFAH1B1 were stained with DAPI (*white*, nucleus) and γH2AX (*green*, DNA DSBs) 4 days post-transfection to visualize DNA damage. Scale bars are 20 μm. *D*, γH2AX foci for individual cells from multiple microscope fields were counted, and cells were grouped by number of foci. *E*, same as panel (*A*), but using RNA-seq data from BT549 cells. *F*, same as panel (*B*), but in BT549 cells. *G* and *H*, same as panels (*C* and *D*), but in BT549 cells. For all data, n = 3, points are technical replicates for each biological replicate, bars are means ± SD. ∗*p* < 0.05, ∗∗∗*p* < 0.001 by unpaired two-tailed *t* test.

A common cause of G2/M arrest is DNA damage, which can lead to significant deleterious effects during mitosis. As cells undergoing mitosis encounter DNA damage, mitotic progression is halted to provide time for activating repair pathways and resolving any damage before continuing (39). Further interrogation of the RNA-seq data described above revealed significant enrichment of DNA repair pathways in siPAFAH1B1 cells, suggesting that cells with reduced expression of *PAFAH1B1* may experience increased DNA damage (Fig. 5*A*). Accumulation of phosphorylated histone H2AX (aka pH2AX or γH2AX) binds to DNA double-strand breaks (DSBs) and recruits repair machinery (40). Silencing *PAFAH1B1* in MDA-MB-231 cells resulted in a ∼2X increase in phosphorylated H2AX (Figs. 5*B* and S3*B*), suggesting that *PAFAH1B1* may be important for maintaining DNA integrity. We also quantified the number of γH2AX foci in each cell using immunohistochemistry (Fig. 5*B*). Cells transfected with the non-silencing control have very low amounts of DNA damage as indicated by the vast majority of cells having ≤5 γH2AX foci/cell (Fig. 5, *C* and *D*). In contrast, *PAFAH1B1* silencing resulted in a much greater proportion of MDA-MB-231 cells with >5 foci/cell. Like the MDA-MB-231 cells, the RNA-seq data from BT549 cells also revealed enrichment of DNA repair pathway genes (Fig. 5*E*). These cells also displayed a robust increase in pH2AX levels (Figs. 5*F* and S3*B*) and γH2AX foci (Fig. 5, *G* and *H*) in response to *PAFAH1B1* suppression. The increase in H2AX phosphorylation and accumulation at foci within both cell lines indicates that *PAFAH1B1* is essential for maintaining DNA integrity in TNBC cells.

### Paclitaxel-resistant cells have an increased dependency on *PAFAH1B1*

**Figure 6:**
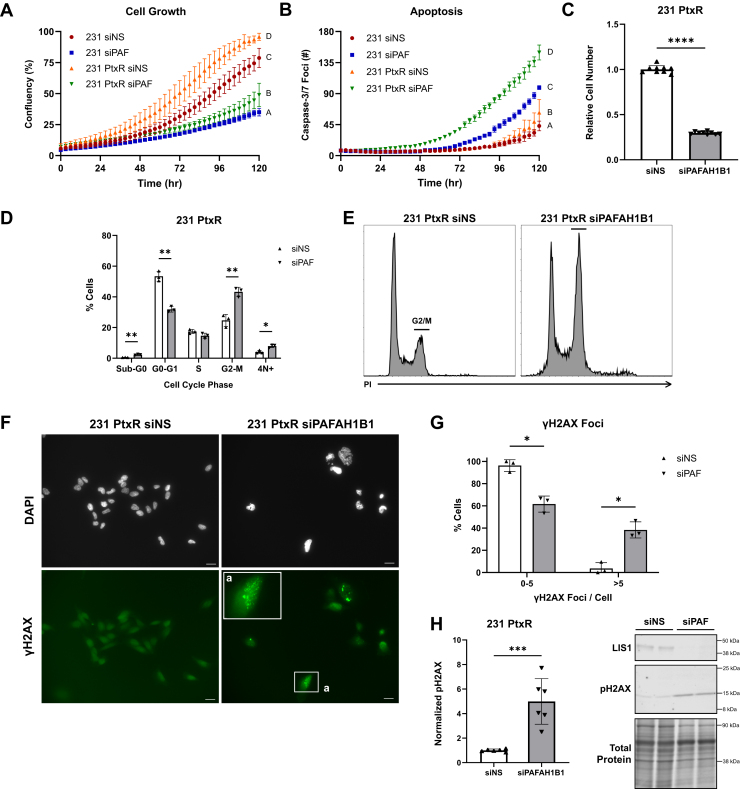
**Paclitaxel-resistant cells have an increased dependency on *PAFAH1B1*.***A* and *B*, MDA-MB-231 parental and paclitaxel-resistant (PtxR) cells were transfected with siNS or siPAFAH1B1 and then tracked over time using the Incucyte Live-Cell Analysis System to measure (*A*) confluency and (*B*) apoptosis with the CellEvent Caspase-3/7 detection reagent. n = 4, points are means ± SD. *C*, MDA-MB-231 PtxR cells transfected with siNS or siPAFAH1B1 were allowed to grow for 5 days. Cell number was quantified using crystal violet staining. n = 3, points are technical replicates for each biological replicate, bars are means ± SD. *D*, MDA-MB-231 PtxR cells transfected with siNS or siPAFAH1B1 were fixed and stained with propidium iodide 3 days after transfection. Flow cytometry revealed an increase in the population of cells in G2/M in response to *PAFAH1B1* silencing. n = 3, points are biological replicates, bars are means ± SD. *E*, representative PI histograms of MDA-MB-231 PtxR cells with or without *PAFAH1B1* silencing. *F*, MDA-MB-231 cells transfected with siNS or siPAFAH1B1 were stained with DAPI (*white*, nucleus) and γH2AX (*green*, DNA DSBs) 4 days after transfection. Scale bars are 20 μm. *G*, γH2AX foci for individual cells from multiple microscope fields were counted, and cells were grouped by number of foci. n = 3, bars are means ± SD. *H*, Western blotting for pH2AX expression at day 5 in *PAFAH1B1*-silenced or siNS-transfected cells. n = 3, points are technical replicates for each biological replicate, bars are means ± SD. ∗*p* < 0.05, ∗∗*p* < 0.01, ∗∗∗*p* < 0.001, ∗∗∗∗*p* < 0.0001 by unpaired two-tailed *t* test. For panels (*A*–*B*), groups with different letters are statistically different from one another (*p*-adj < 0.05) by one-way ANOVA and Sidak’s multiple comparisons test.

As indicated earlier, TNBC responds well to taxanes at the onset of treatment, but acquired resistance is common (41). Mitotic delays and DNA DSBs are consequences of taxane treatment that drive tumor cell death (42, 43). The above studies indicated that *PAFAH1B1* controls the timing of mitosis as well as prevents DSB accumulation; thus, we postulated that paclitaxel-resistant (PtxR) cells may be particularly dependent on *PAFAH1B1* for growth and survival to a greater extent than parental cells. To assess this possibility, we used MDA-MB-231 cells that are resistant to paclitaxel that we previously generated (44), and conducted a side-by-side comparison of parental and PtxR cells with or without *PAFAH1B1* silencing. Confluency and apoptosis were quantified over time. As expected, parental cells with reduced *PAFAH1B1* levels grew more slowly than cells transfected with the non-silencing control siNS (Fig. 6*A*). They also displayed an increase in apoptosis, as evidenced by a greater number of caspase-3/7 foci (Fig. 6*B*). This is consistent with the cell death observed with live-cell imaging performed at an earlier timepoint (Fig. 4*G*) and indicates that the incidence of cell death increases with prolonged reduction of *PAFAH1B1* expression. PtxR cells grew faster than parental cells transfected with siNS but had a comparable level of cell death. Silencing *PAFAH1B1* in the PtxR cells also reduced cell growth, to an extent similar to the parental cells, indicating that *PAFAH1B1* dependency is maintained with the acquisition of Ptx resistance (Fig. 6*A*). Most importantly, the onset of apoptosis in the siPAFAH1B1 PtxR cells (at ∼36 h) was markedly earlier and to a greater extent than the parental cells (at ∼72 h) (Fig. 6*B*). Using crystal violet staining as an orthogonal approach confirmed that silencing *PAFAH1B1* decreases cell number 5 days after transfection compared to cells transfected with the siNS control (Fig. 6*C*). Cell cycle analysis of the PtxR cells further revealed a substantial increase in the proportion of cells in G2/M, as well as increases in the sub-G0 and 4N+ populations in response to *PAFAH1B1* silencing (Fig. 6*D*). The striking magnitude of G2/M arrest in the PtxR cells upon *PAFAH1B1* silencing was far greater than that observed in the parental cells (*p*-adj < 0.05, Figs. 6, *E* and 4, *D*). We further investigated whether PtxR cells also exhibit increased DNA damage in response to reduced *PAFAH1B1* expression. Indeed, both the number of γH2AX foci and levels of phosphorylated H2AX protein were increased in response to siPAFAH1B1 transfection compared to siNS-transfected cells that are PtxR (Fig. 6, *F*–*H*). Taken together, these data indicate that paclitaxel resistance confers heightened dependency on *PAFAH1B1*, with its loss leading to reduced cell growth, greater accumulation of cells in the G2/M phase of the cell cycle, and increased cell death compared to parental cells.

### Sensitivity to paclitaxel is increased in parental and resistant TNBC cells upon *PAFAH1B1* suppression

**Figure 7:**
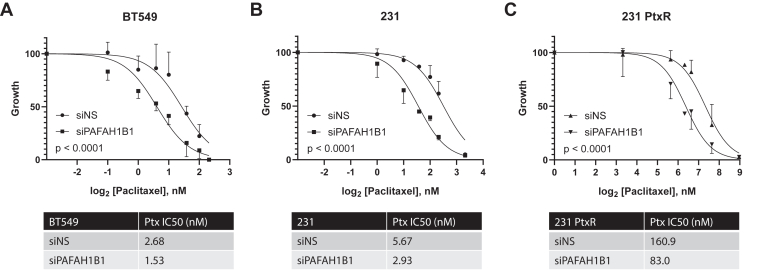
**Sensitivity to paclitaxel is increased in parental and resistant TNBC cells upon *PAFAH1B1* suppression.***A*, Ptx dose-response curves measuring BT549 cell number were completed 8 days after transfection with siNS or siPAFAH1B1 and treatment with Ptx. Each curve is normalized against its own vehicle-treated control. Calculated IC50 values are provided below the graph. *B*, same as panel (*A*), but for parental MDA-MB-231 cells. *C*, same as panel (*B*), but for MDA-MB-231 paclitaxel-resistant (PtxR) cells. For all data, n = 3, points are means ± SD. Curves were fit using nonlinear regression and compared using the extra sum-of-squares F test.

Having observed the strong dependency of parental and PtxR cells on *PAFAH1B1*, we then evaluated the impact of *PAFAH1B1* silencing on paclitaxel responsiveness. Cells were transfected with siPAFAH1B1 or the siNS control and treated with Ptx for 8 days. Given the growth reduction in *PAFAH1B1*-silenced cells, we normalized growth values of siNS or siPAFAH1B1 to their own vehicle-treated controls when quantifying drug response. In response to siPAFAH1B1, BT549 cells exhibited ∼1.75-fold reduction in the IC50 for Ptx (2.68 nM to 1.53 nM, Fig. 7*A*). Similarly, parental and PtxR MDA-MB-231 cells also exhibited ∼2-fold reductions in the Ptx IC50 upon *PAFAH1B1* silencing, with parental MDA-MB-231 cells shifting from 5.67 nM to 2.93 nM, and MDA-MB-231 PtxR cells going from 160.9 nM to 83.0 nM (Fig. 7, *B* and *C*). These data suggest that targeting LIS1/*PAFAH1B1* may represent a novel strategy for improving the responsiveness of TNBC cells to taxanes.

## Discussion

Identifying novel vulnerabilities of TNBC will be essential for developing new therapeutic approaches for this disease. The data presented herein revealed that *PAFAH1B1* is a previously undescribed regulator of mitotic progression and DNA integrity in TNBC models. With suppression of *PAFAH1B1*, TNBC cells exhibit stunted growth and a G2/M arrest associated with increased DNA DSBs and apoptosis. These defects are magnified in PtxR TNBC cells, implicating LIS1/*PAFAH1B1* as a heightened vulnerability in the context of Ptx resistance that could be exploited therapeutically. Notably, while LIS1 protein expression was not increased in the PtxR cells (Fig. S4), these cells appear to have an increased reliance on this protein. Mechanistically, it is unclear what underlies this elevated dependency on *PAFAH1B1*, and further investigation is needed. However, given the established mitotic roles of *PAFAH1B1* in neural stem and progenitor cells, it is likely that TNBC cells, and especially PtxR cells, have co-opted a primitive feature of their development to use *PAFAH1B1* as a safety net when faced with aberrant mitoses and DNA damage (16, 45). In support of this, a combination *in vitro*/*in vivo* CRISPR screen using a PtxR TNBC cell line identified 34 genes associated with paclitaxel response, several of which were associated with stemness (46). Furthermore, PtxR TNBC cells have been shown to be dependent on other regulators of proper mitosis and cell cycle progression. PRMT5 inhibition increased chromosome missegregation and multipolar spindle formation only in PtxR, but not parental, TNBC cells due to alternative splicing and subsequently reduced expression of AURKB, a key mitotic kinase important for chromosome alignment and segregation (47). Additionally, miR-153-5p was reported to regulate paclitaxel sensitivity in PtxR TNBC cells by binding to CDK1 and subsequently inducing G2/M arrest (48). As such, PtxR cells most likely depend on *PAFAH1B1* for similar reasons, ensuring mitotic fidelity and continued cell cycle progression.

The increased DNA DSBs that occur upon reducing *PAFAH1B1* expression further reveal a novel function of *PAFAH1B1* in maintaining genome integrity. *PAFAH1B1*/LIS1 has been reported to work with dynein to prevent multipolar spindles and ensure proper chromosome segregation (49). Thus, the increase in DNA DSBs may be the result of non-amphitelic attachment of kinetochores to microtubules or the presence of multipolar spindles, both of which would be expected to result in significant damage to DNA. Consistent with other cancer models, suppressing *PAFAH1B1* in TNBC cells led to centrosome amplification as well as disorganized metaphase plates suggestive of multipolar spindles that are known to increase DNA damage during mitosis (50). While multipolar spindles and aberrant chromosomal segregation are likely the major contributors to the increase in breaks observed herein, there are other possible causes to consider. For example, LIS1 may exert a more direct role in the formation or repair of DNA DSBs. Additionally, multiple studies have shown that cells accumulate DNA damage merely by spending more time in mitosis, regardless of cell type or method of pharmacological or genetic manipulation used to prolong mitosis (42, 51, 52). As such, further study is needed to determine the extent to which these factors may contribute to the observed DNA damage. Additionally, some cells with *PAFAH1B1* silencing appeared to exhibit abnormal nuclei, such as micronuclei and dysmorphic nuclei (Fig. 5, *C* and *G*). Such phenotypes are associated with chromosome instability (CIN), a common hallmark of many cancers that can increase cancer cell fitness, but when occurring in excess ultimately induces intolerable DNA damage and cell death (53). CIN-inducing agents such as paclitaxel target this vulnerability by increasing CIN to excessive levels, causing cell death (54, 55). The nuclear atypia and CIN observed following *PAFAH1B1* suppression may represent one mechanism by which taxane sensitivity is enhanced.

In the Broad DepMap, *PAFAH1B1* is classified as a common essential gene, a designation that, by definition, ranks it among the top genes whose loss perturbs growth in at least 90% of all cell lines tested. This classification could be interpreted as an indicator of poor suitability for drug development. However, many common essential genes have been shown to be highly effective therapeutic targets. Most notably, CDK4 and DNMT1 are inhibited by FDA-approved drugs for the treatment of luminal breast cancers and bone marrow diseases, respectively, despite being common essential genes (56, 57). This is likely due to modest rather than complete inhibition of these enzymes by such agents. Importantly, we found that reducing *PAFAH1B1* expression did not impact non-transformed mammary epithelial cells. These data suggest that reducing, rather than eliminating, the function of *PAFAH1B1*/LIS1 may be a viable therapeutic approach for the treatment of TNBC and possibly other cancers that depend on this protein. Notably, motor proteins have progressively gained attention as potential anti-cancer targets. Integrated analysis of 19 studies comprising patients with solid tumors revealed that ∼50% of tumors had copy number alterations of at least one member of the dynein or kinesin families, and these alterations correlated with worse overall survival (58). Both families of motor proteins play important roles in mitosis, and multiple studies have explored targeting their activity using small-molecule inhibitors. However, the majority suffer from a lack of potency or poor efficacy, while some cause hematological toxicity (59, 60, 61). In contrast, a regulator of dynein, specifically LIS1, may represent a more effective and less toxic therapeutic target by modulating specific dynein functions. Interestingly, genetic manipulation of other dynein regulators elicits fewer or milder cell-cycle-related phenotypes in HeLa cells when compared to the effects of LIS1/*PAFAH1B1* loss, suggesting that LIS1 may be more important for the mitotic functions of dynein, especially in cancer (62).

LIS1 controls dynein activity without having an enzymatic function. Thus, designing an inhibitor of LIS1 will most likely require targeting its interface with dynein. Disrupting protein–protein interactions (PPIs) with small molecules can be highly challenging primarily due to the absence of a defined binding pocket. However, there have been significant advancements in the past decade that suggest that specific interfaces involved in PPIs are more targetable than previously expected (63). One particularly relevant example is the interaction between the histone methyltransferase MLL1, and WDR5 (WD repeat-containing protein 5). MLL1 is the catalytic subunit of a complex that also includes ASH2L, RBBP5, and WDR5. This multimer methylates H3K4, enhancing chromatin accessibility (64). Translocations that give rise to MLL1 fusion proteins can cause acute leukemias due to sustained homeobox gene expression and MYC recruitment to chromatin (65, 66). Initial studies using peptidomimetics successfully disrupted the interaction between WDR5 and MLL1, setting the stage for the development of small-molecule inhibitors that have performed well in the preclinical setting (67, 68). The structural similarities between WDR5 and LIS1 in their binding interfaces provide strong support for the targetability of LIS1 and disruption of its interaction with dynein. Notably, recent studies utilizing cryo-EM have generated high-resolution structures of the LIS1 and dynein complex, providing a foundation for the development of peptidomimetics and subsequent potential small-molecule inhibitors of this interaction (25, 69).

In summary, we found that LIS1/*PAFAH1B1*, a regulator of neurogenesis, is also essential for TNBC cell growth. Given the developmental relationship between breast epithelium and neuroectoderm, this may be due to a reliance of TNBC on primitive pathways controlling proliferation. Moreover, sustained expression of *PAFAH1B1* was necessary for resisting paclitaxel-induced growth suppression and cell death. While paclitaxel remains a mainstay chemotherapy for TNBC and other cancers, resistance remains a major clinical hurdle, and extending its efficacy will require approaches that circumvent and combat resistance. The data presented herein suggest that targeting LIS1 may provide an approach to improve paclitaxel responsiveness in TNBC.

## Materials and methods

### Cell culture and reagents

MDA-MB-231, HCC38, BT549, MCF10A, and MCF12A cell lines were obtained from the American Type Culture Collection (ATCC, Manassas, VA). MDA-MB-231, HCC38, and BT549 cells were grown in RPMI 1640 media supplemented with 10% FBS and 1% Pen-Strep. Media for BT549 cells was additionally supplemented with 0.8 μg/ml insulin. MCF10A and MCF12A cells were grown in DMEM/F-12 media supplemented with 0.1 μg/ml cholera toxin, 0.5 μg/ml hydrocortisone, 10 μg/ml insulin, 20 ng/ml EGF, 5% horse serum, and 1% Pen-Strep. Paclitaxel-resistant (PtxR) cells were generated by treating MDA-MB-231 cells with escalating concentrations of Ptx over the course of several months. Cells were treated for 4 days and given a drug holiday for 3 days before re-treatment. All cells were maintained at 37 °C in 5% CO_2_ and tested monthly for *mycoplasma* contamination using the MycoAlert Plus *Mycoplasma* Detection Kit (Lonza, LT07-703) or *Mycoplasma* PCR Detection Kit (Abcam, ab289834). Cell lines were authenticated using STR analysis in 2022 or 2024 (Labcorp). Ptx was obtained from LC Laboratories (P-9600).

### siRNA transfection

Transient silencing of gene expression was achieved using forward transfection. siRNA targeting *PAFAH1B1* or control, non-targeting siRNA was diluted in OptiMEM to a final concentration of 100 nM, mixed with Lipofectamine 2000 that was diluted in OptiMEM to a final 1:90 ratio, and allowed to incubate for 20 min at RT. Cells were washed with 1X PBS and placed in fresh OptiMEM. The siRNA/Lipo/OptiMEM mixture was then added dropwise to cells. After 6 h, the mixture was aspirated and replaced with complete media. The following siRNAs were purchased from Horizon Discovery and used in this study:

**Table:** 

siRNA name	Catalog number	Target sequence(s)
ON-TARGETplus Human PAFAH1B1 SMARTpool (comprised of siRNA #6-9)	L-010330-00	#6: CAAUUAAGGUGUGGGAUUA #7: UGAACUAAAUCGAGCUAUA #8: GGAGUGCCGUUGAUUGUGU #9: UGACAAGACCCUACGCGUA
ON-TARGETplus Human PAFAH1B1 siRNA #6	J-010330-06	CAAUUAAGGUGUGGGAUUA
ON-TARGETplus Human PAFAH1B1 siRNA #9	J-010330-09	UGACAAGACCCUACGCGUA
siGENOME Non-Targeting Control siRNA #5 (siNS)	D-001210-05	UGGUUUACAUGUCGACUAA

### Western blot analysis

Cells were washed in 1X PBS and lysed in RIPA buffer containing 1% protease inhibitor cocktail (Millipore Sigma, P8340) and 1 mM sodium orthovanadate. After shaking for 10 min at 4 °C, cells were scraped off the plate, transferred to an Eppendorf tube, incubated on ice for 30 min with occasional vortexing, and then centrifuged at 10,000*g* for 10 min. Supernatants were stored at −20 °C. Protein concentration was determined using the Bradford protein assay (Bio-Rad, #5000006) with absorbance values at 600 nm using a Promega GloMax Explorer Plate Reader. 50 to 75 μg of protein lysate was mixed 1:1 with 2X Laemmli buffer containing β-mercaptoethanol, boiled at 100 °C for 10 min, and loaded onto 4 to 20% Tris-Glycine mini protein gels (Invitrogen, XP04200BOX). Gels were run at 175 V for 1 h and transferred onto an Immobilon-FL PVDF membrane (Millipore Sigma, IPFL00010) at 35 V overnight. Membranes were stained for total protein using REVERT (LI-COR, 926-11021) and then imaged on a LI-COR Odyssey CLx. After destaining, membranes were blocked in 5% BSA/TBS with shaking for 1 h at RT. Membranes were incubated with primary antibody diluted 1:1000 in 5% BSA/TBS with 0.1% Tween-20 at 4 °C overnight, incubated in secondary antibody diluted 1:15,000 in 5% milk/TBS-T with 0.02% SDS for 1 h at RT in the dark, and imaged on a LI-COR Odyssey CLx. Protein levels were quantified using Image Studio v5.2 and normalized to total protein. The following antibodies were used in this study: anti-LIS1 (CST, 12453); anti-pH2AX (CST, 80312); IRDye 800CW anti-rabbit secondary (LI-COR, 926-32211); IRDye 800CW anti-mouse secondary (LI-COR, 926-32210). Primary antibodies were validated using siRNA and blotting for the targeted protein.

### RNA isolation and cDNA synthesis

RNA was isolated using TRIzol (Invitrogen, 15596018) and treated with DNase (Invitrogen, AM1906), following the manufacturer’s protocol. RNA concentration and purity was measured using a NanoDrop One (ThermoFisher Scientific). cDNA was generated using random primers (Invitrogen, 48190011), dNTPs (Invitrogen, 10297-018), and SuperScript IV reverse transcriptase (Invitrogen, 18090-050), following the manufacturer's protocol.

### Quantitative PCR

Quantitative real-time PCR was performed using a StepOnePlus Real-Time PCR System (ThermoFisher Scientific). Gene expression was calculated relative to *GAPDH* or *TBP* using the ΔΔCT method and normalized to control siNS samples. The following TaqMan probes were purchased from ThermoFisher Scientific and used in this study: *PAFAH1B1* (Hs00181182_m1); *GAPDH* (Hs02758991_g1); *TBP* (Hs99999910_m1).

### Cell growth

Cells were transfected with siPAFAH1B1 or siNS as described above. Live cell number was determined using either trypan blue exclusion and a Countess II FL Automated Cell Counter, or Crystal violet staining and a Promega GloMax Explorer Plate Reader. For both methods, cell number was normalized relative to control siNS cells. For crystal violet staining, cells were washed with 1X PBS, stained with 0.05% crystal violet/PBS for 20 min, washed with water, and allowed to dry overnight. 10% acetic acid was added to solubilize the stain, incubated for 15 min with gentle shaking, and aliquoted into separate wells for technical replicates. Absorbance at 600 nm was determined using the plate reader.

### RNA-sequencing

MDA-MB-231, HCC38, and BT549 cells were transfected with siPAFAH1B1 or siNS in quadruplicate as described above, and RNA was isolated 72 h later using the RNeasy Plus Micro Kit (Qiagen, 74034). Confirmation of knockdown was performed using RT-PCR as described above. Library preparation, sequencing, and alignment were performed by Novogene Corporation Inc., using an Illumina NovaSeq 6000 to generate paired-end 150 bp reads that were mapped to hg38. Differentially expressed genes (DEGs) were identified using the R package DESeq2, with a cutoff of Benjamini-Hochberg *p*-adj < 0.05 (70). Venn diagrams comparing DEG overlap between cell lines were generated using the R package eulerr (71). Heatmaps comparing DEG expression across cell lines were generated using the R package ComplexHeatmap (72).

### Gene set enrichment

All DEG lists were ranked by Log2FC, and Gene Set Enrichment Analysis (GSEA) was performed using the GSEA desktop application for individual cell line RNA-seq data or using the R package clusterProfiler for TCGA BRCA patient data (73, 74, 75). All Gene Ontology (GO) analyses were performed using clusterProfiler and DEG lists split into separate up and down lists using a cutoff of absolute Log2FC > 0.5. To remove redundant GO terms before visualization, the simplify function was used with a threshold of 0.7.

### Public database analyses

Gene expression and survival data for TCGA BRCA patients was downloaded using cBioPortal (76, 77, 78). Patients were binned into quartiles based on *PAFAH1B1* expression, and the high *versus* low quartiles were compared for differences in overall survival using the log-rank test. The list of DEGs (*p*-adj < 0.05) between the high *versus* low *PAFAH1B1* quartiles was also downloaded from cBioPortal and filtered for genes associated with proliferation, identified by Venet *et al.* (35, 79). Using the full TCGA BRCA cohort, multivariate Cox proportional-hazards analysis was performed using the R packages survival and survminer to calculate a hazard ratio based on *PAFAH1B1* expression, setting *ESR1*, *MKI67*, and *ERBB2* (HER2) expression as covariates.

CRISPR essentiality data was downloaded from the BioGRID Open Repository of CRISPR Screens (80). The dataset generated by Meyers *et al.* was filtered for breast cancer cell lines, and dependency rankings for *PAFAH1B1* and other dynein regulators were compared (81). Data generated by Behan *et al.* and Sack *et al.* were included when tabulating rankings to facilitate comparisons with luminal breast cancer and normal breast epithelial cell lines, respectively (82, 83).

### Live cell imaging

MDA-MB-231 cells were transfected with siPAFAH1B1 or siNS as described above and plated the following day in a six or 24 well plate. Cells were then allowed to rest for 24 h before being moved into an Incucyte S3 Live-Cell Analysis System (Sartorius). Phase-contrast images were captured at 20× every 20 min for 3 days. The start of mitosis for a cell was designated by identifying the timepoint at which the cell had achieved a rounded morphology and then starting the time 20 min earlier. The end of mitosis for a cell was designated as the timepoint at which both daughter cells had re-adhered to the plate. Mitotic fates were classified as follows: exit and divide (two daughter cells), exit and die (at least one daughter cell dies shortly after completing mitosis), die in mitosis (cell dies after formation of the metaphase plate), failed cytokinesis (cell undergoes mitosis but fails to form two daughter cells and re-adheres to the plate), prolonged interphase (cell fails to divide again after previous mitosis), or aberrant mitosis (cell with disorganized metaphase plate, presence of chromatin bridge, or any other visible abnormalities).

### Flow cytometry

HCC38, MDA-MB-231, and MDA-MB-231 PtxR cells were transfected with siPAFAH1B1 or siNS as described above. Cells were trypsinized and centrifuged at 500*g* for 5 min. The cell pellet was resuspended in 1X PBS, centrifuged at 1000*g* for 5 min, fixed in ice-cold 70% ethanol, and stored at 4 °C. Cells were then centrifuged at 1000*g* for 5 min, washed twice with 1% BSA/PBS, and resuspended in 250 μl PBS. 250 μl 2x PI/RNase solution (0.1% Triton X-100/PBS with 0.2 mg/ml RNase A and 20 μg/ml propidium iodide) was added, and cells were incubated at 37 °C for 30 min in the dark. Cells were filtered with a 35 μm cell strainer and evaluated using an Attune Nxt, BD LSRFortessa, or BD FACSymphony A5 SE. Unstained controls were used to optimize FSC and SSC voltages, and stained controls were used to optimize PI voltage. Using FlowJo v10 software, cells were gated to exclude cell debris and doublets, and the remaining population was analyzed to determine the proportion of cells in each stage of the cell cycle.

### Immunofluorescence microscopy

BT549, MDA-MB-231, and MDA-MB-231 PtxR cells were transfected with siPAFAH1B1 or siNS as described above and plated the following day on glass coverslips. 24 h later, control coverslips (from each transfection group) were treated with 10 μM etoposide as a positive control for DNA damage and validation of antibody specificity. 48 h later, coverslips were washed with 1X PBS, fixed in 4% paraformaldehyde for 10 min at RT, and then stored in 1X PBS at 4 °C. Cells on coverslips were permeabilized in 0.1% Triton X-100/PBS for 10 min at RT and washed with 1X PBS. For γH2AX staining, coverslips were then incubated in Texas Red-X phalloidin (Invitrogen, T7471) for 20 min at RT in the dark. After washing with 1X PBS, coverslips were incubated in Alexa Fluor 488 anti-γH2AX (BD Biosciences, 560445) overnight at 4 °C in the dark. For centrosome staining, after permeabilization, coverslips were incubated in Alexa Fluor 488 anti-γ-tubulin (Abcam, ab205475) overnight at 4 °C in the dark. After washing with 1X PBS, coverslips were mounted on slides using Prolong Gold Mountant with DAPI (Invitrogen, P36962) and allowed to dry in the dark. Slides were imaged at 40x or 60x on a Keyence BZ-X810 or Leica DMS200 microscope. γH2AX or γ-tubulin foci were quantified for each cell and then cells were binned into groups based on foci number.

### Apoptosis

To measure apoptosis over time, the CellEvent Caspase-3/7 Green Detection Reagent (Invitrogen, C10423) was used while imaging cells with an Incucyte S3. MDA-MB-231 and MDA-MB-231 PtxR cells were transfected with siPAFAH1B1 or siNS as described above and plated the following day in a 96 well plate. Cells were then allowed to rest for 24 h before treatment with one of the following: 0.1% DMSO, 500 nM staurosporine (SSP, positive control), 0.1% DMSO + 2 μM Caspase-3/7 reagent, or 500 nM SSP + 2 μM Caspase-3/7 reagent. Cells were moved into the Incucyte S3 and imaged at 10× every 3 h for 6 days. Phase-contrast images were captured for measuring confluency, and green fluorescence images were captured for counting apoptotic cells with caspase-3/7 foci.

### Dose–response curves

BT549, MDA-MB-231, and MDA-MB-231 PtxR cells were transfected with siPAFAH1B1 or siNS as described above and plated the following day in a 24 well plate. Cells were then allowed to rest for 24 h before being treated with 0.1% DMSO or Ptx. Cells were then imaged with an Incucyte S3 at 10× every 3 h for 8 days. At the midpoint of the experiment, cells were retreated with fresh media and drug. Confluency over time was monitored to ensure vehicle-treated control cells did not reach 100% confluency before the endpoint. Confluency was normalized to each group’s own vehicle-treated cells to account for the reduced cell growth with *PAFAH1B1* silencing.

### Statistical analyses

Statistical analyses were performed using two-tailed Student’s *t* test for all data, unless otherwise stated. The multivariate Cox proportional-hazards model using TCGA patient data was analyzed using the score (log-rank) test. Apoptosis and confluency data were analyzed using a one-way ANOVA, followed by the Sidak correction for multiple comparisons. Dose-response curves were analyzed by fitting nonlinear curves with a constrained Hill Slope for each cell line and using the extra sum-of-squares F test. Unless noted otherwise, all data are represented as means with SD of three independent experiments, each completed in duplicate or triplicate.

## Data availability

The RNA-seq data generated in this study are publicly available at NCBI GEO (GEO accession number GSE285320).

## Supporting information

This article contains supporting information.

## Conflict of interest

The authors declare that they have no conflicts of interest with the contents of this article.
