# Supplementary material for: The neural stem cell gene PAFAH1B1 controls cell cycle progression, DNA integrity, and paclitaxel sensitivity of triple-negative breast cancer cells
Source: J Biol Chem. 2025 May 14;301(6):110235. doi: 10.1016/j.jbc.2025.110235 (PMC12192685; doi:10.1016/j.jbc.2025.110235)
Supplement: Fig. S1 [file mmc1.pdf]

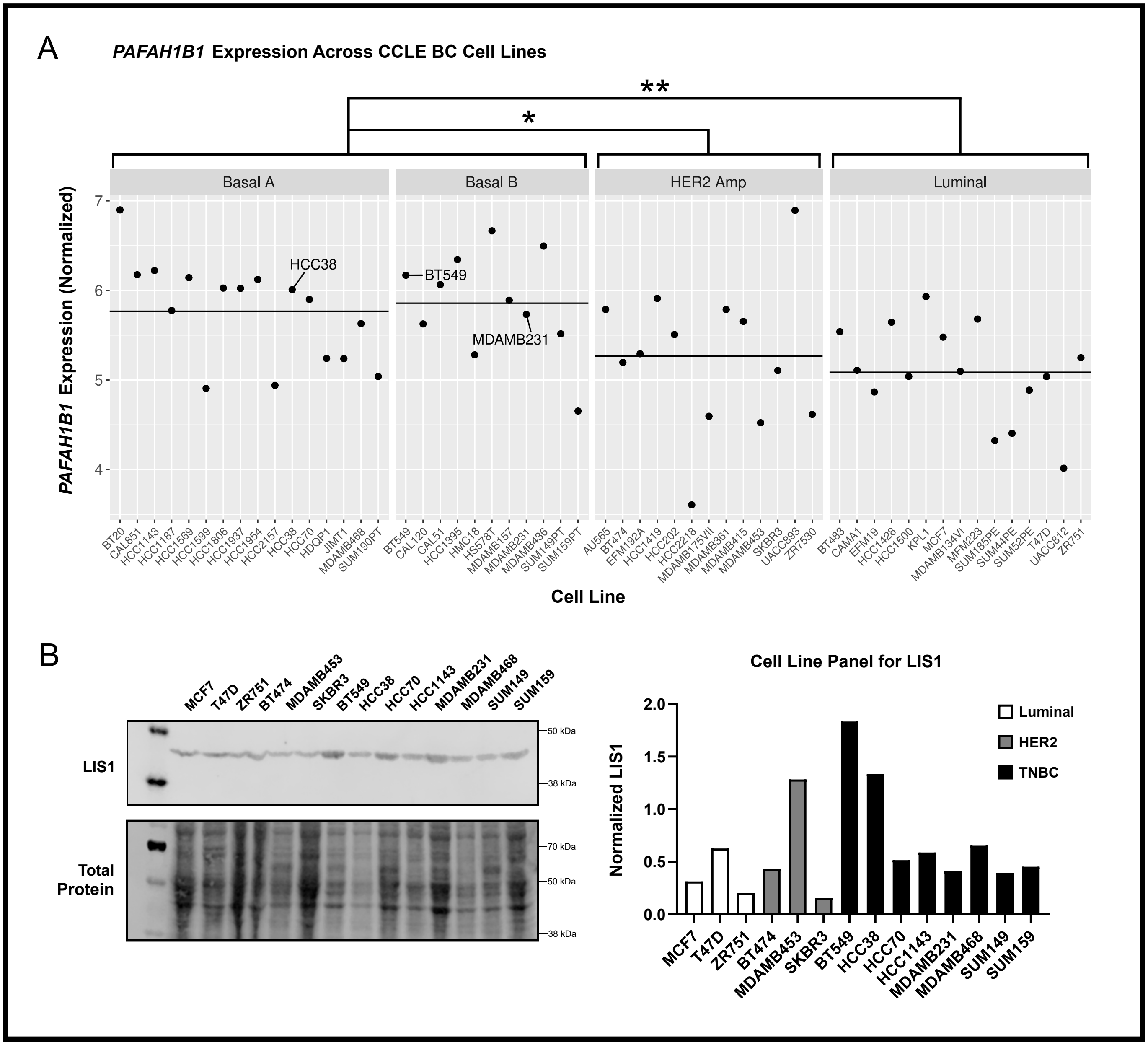

Supplemental Figure 1. *PAFAH1B1* mRNA and protein expression are highest in TNBC (Basal A and Basal B) cell lines.

A) Normalized *PAFAH1B1* expression values across all CCLE breast cancer cell lines, grouped by subtype. Horizontal lines indicate mean value for that subtype. *p-adj < 0.05, **p-adj < 0.01 by the Wilcoxon test and Benjamini-Hochberg correction. B) Western blotting for LIS1 expression in a panel of breast cancer cell lines. Bars are colored by subtype.
