# Supplementary material for: The neural stem cell gene PAFAH1B1 controls cell cycle progression, DNA integrity, and paclitaxel sensitivity of triple-negative breast cancer cells
Source: J Biol Chem. 2025 May 14;301(6):110235. doi: 10.1016/j.jbc.2025.110235 (PMC12192685; doi:10.1016/j.jbc.2025.110235)
Supplement: Fig. S2 [file mmc2.pdf]

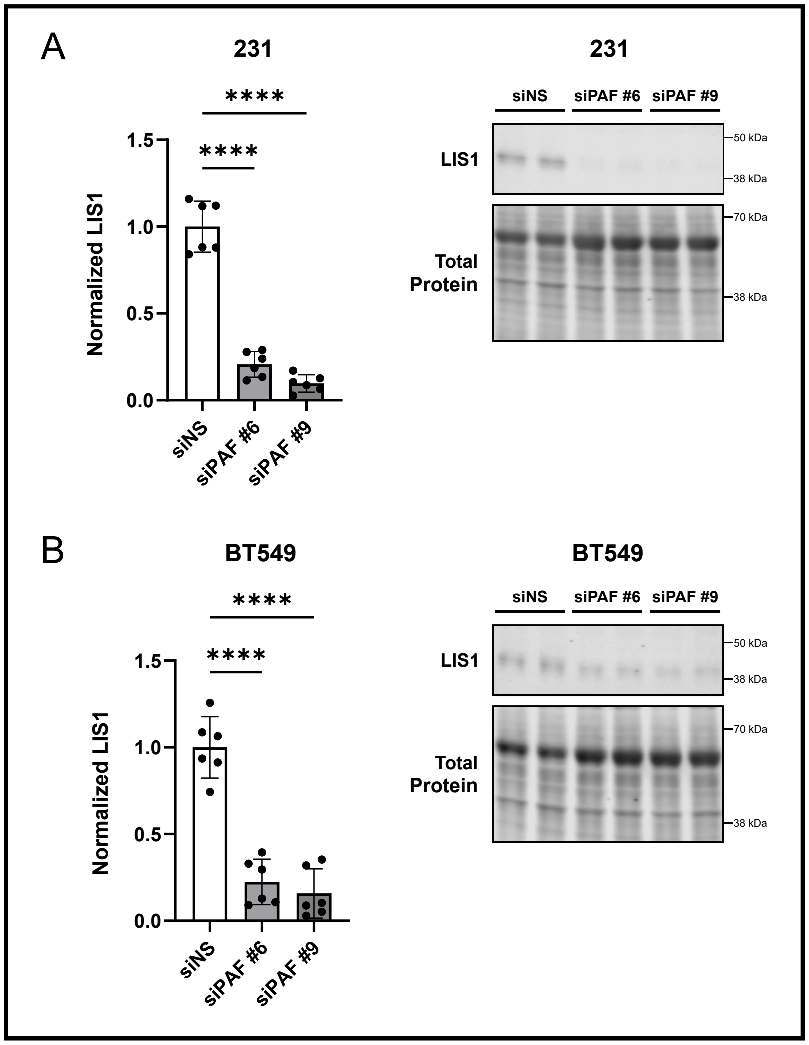

Supplemental Figure 2. LIS1 protein levels are suppressed three days after transient transfection with siPAFAH1B1.
A) Western blotting for LIS1 expression in MDA-MB-231 cells three days following transfection with siNS or individual siRNAs targeting *PAFAH1B1* (siPAF #6 or #9). Representative western blot is shown on the right. B) Same as A, but in BT549 cells. For all data, n=3, points are technical replicates for each biological replicate, bars are means +/- SD. ****$p < 0.0001$ by unpaired two-tailed t-test.
