# Supplementary material for: The neural stem cell gene PAFAH1B1 controls cell cycle progression, DNA integrity, and paclitaxel sensitivity of triple-negative breast cancer cells
Source: J Biol Chem. 2025 May 14;301(6):110235. doi: 10.1016/j.jbc.2025.110235 (PMC12192685; doi:10.1016/j.jbc.2025.110235)
Supplement: Fig. S3 [file mmc3.pdf]

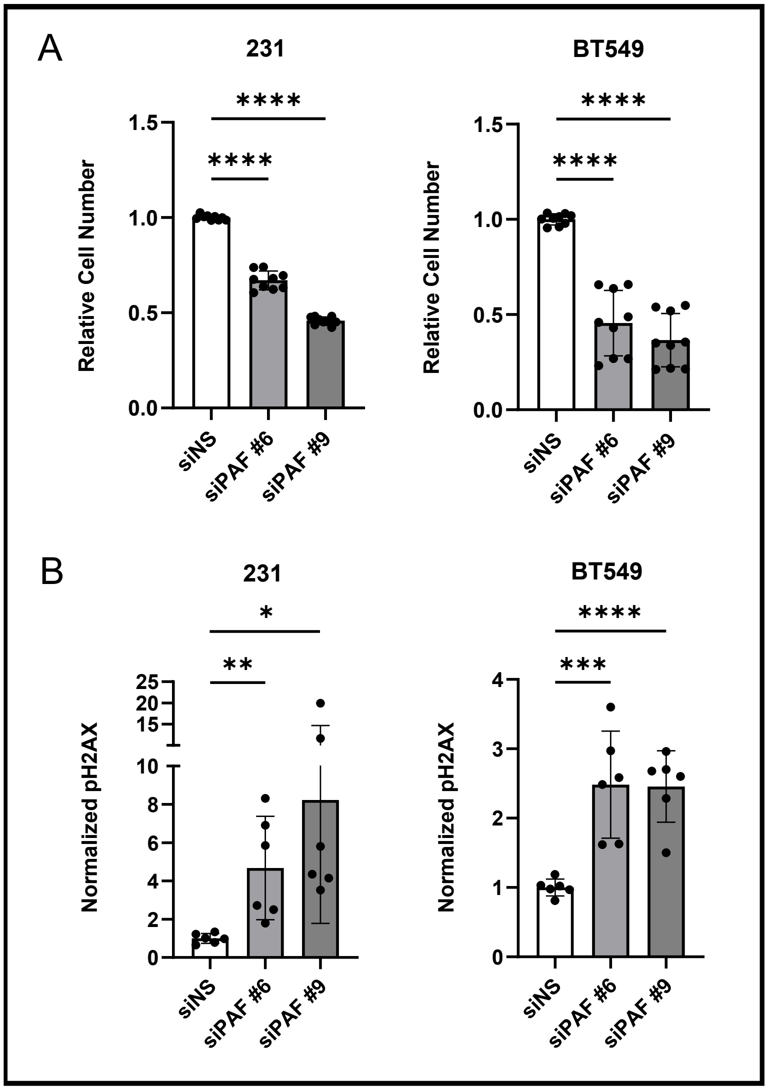

Supplemental Figure 3. *PAFAH1B1* suppression using multiple siRNAs reduces cell growth and increases DNA damage.
A) MDA-MB-231 (left) and BT549 (right) cell number five days after transfection with siNS or individual siRNAs targeting *PAFAH1B1* (siPAF #6 or #9). B) pH2AX expression observed five days after transfection of MDA-MB-231 (left) and BT549 (right) cells. For all data, n=3, points are technical replicates for each biological replicate, bars are means +/- SD. *p < 0.05, **p < 0.01, ***p < 0.001, ****p < 0.0001 by unpaired two-tailed t-test.
