# Supplementary material for: The neural stem cell gene PAFAH1B1 controls cell cycle progression, DNA integrity, and paclitaxel sensitivity of triple-negative breast cancer cells
Source: J Biol Chem. 2025 May 14;301(6):110235. doi: 10.1016/j.jbc.2025.110235 (PMC12192685; doi:10.1016/j.jbc.2025.110235)
Supplement: Fig. S4 [file mmc4.pdf]

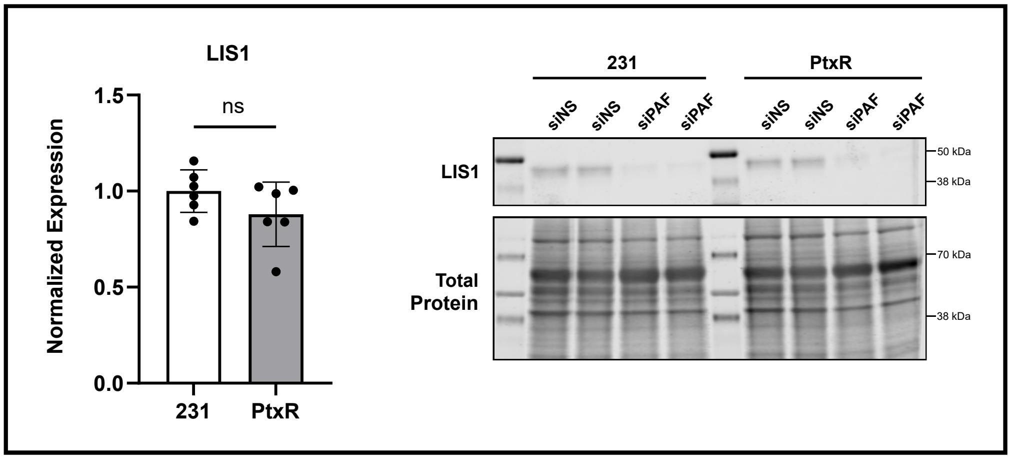

Supplemental Figure 4. LIS1 expression does not differ between parental and PtxR cells.

Western blotting for LIS1 expression in parental and paclitaxel-resistant (PtxR) MDA-MB-231 cells. n=3, points are technical replicates for each biological replicate, bars are means +/- SD. ns, not significant, by unpaired two-tailed t-test. Representative western blot is shown on the right.
